# dPCR: A Technology Review

**DOI:** 10.3390/s18041271

**Published:** 2018-04-20

**Authors:** Phenix-Lan Quan, Martin Sauzade, Eric Brouzes

**Affiliations:** 1 Department of Biomedical Engineering, Stony Brook University, Stony Brook, NY 11794, USA; phuong-lan.quan@stonybrook.edu (P.-L.Q.); martin.sauzade@gmail.com (M.S.); 2 Laufer Center for Physical and Quantitative Biology, Stony Brook University, Stony Brook, NY 11794, USA

**Keywords:** absolute quantification, arrays of microwells, digital PCR, dPCR, droplet microfluidics, microfluidics, microfluidic chambers, microfluidic technologies, on-chip valves, partitioning, quantitative real-time PCR, qPCR, real-time PCR

## Abstract

Digital Polymerase Chain Reaction (dPCR) is a novel method for the absolute quantification of target nucleic acids. Quantification by dPCR hinges on the fact that the random distribution of molecules in many partitions follows a Poisson distribution. Each partition acts as an individual PCR microreactor and partitions containing amplified target sequences are detected by fluorescence. The proportion of PCR-positive partitions suffices to determine the concentration of the target sequence without a need for calibration. Advances in microfluidics enabled the current revolution of digital quantification by providing efficient partitioning methods. In this review, we compare the fundamental concepts behind the quantification of nucleic acids by dPCR and quantitative real-time PCR (qPCR). We detail the underlying statistics of dPCR and explain how it defines its precision and performance metrics. We review the different microfluidic digital PCR formats, present their underlying physical principles, and analyze the technological evolution of dPCR platforms. We present the novel multiplexing strategies enabled by dPCR and examine how isothermal amplification could be an alternative to PCR in digital assays. Finally, we determine whether the theoretical advantages of dPCR over qPCR hold true by perusing studies that directly compare assays implemented with both methods.

## 1. Introduction: Quantification of Nucleic Acids by Quantitative PCR and Digital PCR

In this section, we present the basic concepts that underlie the quantification of nucleic acids by digital and quantitative real-time PCR. An experimental comparison between the two methods will be detailed in Section 7.

### 1.1. PCR and Quantitative PCR

**Figure 1:**
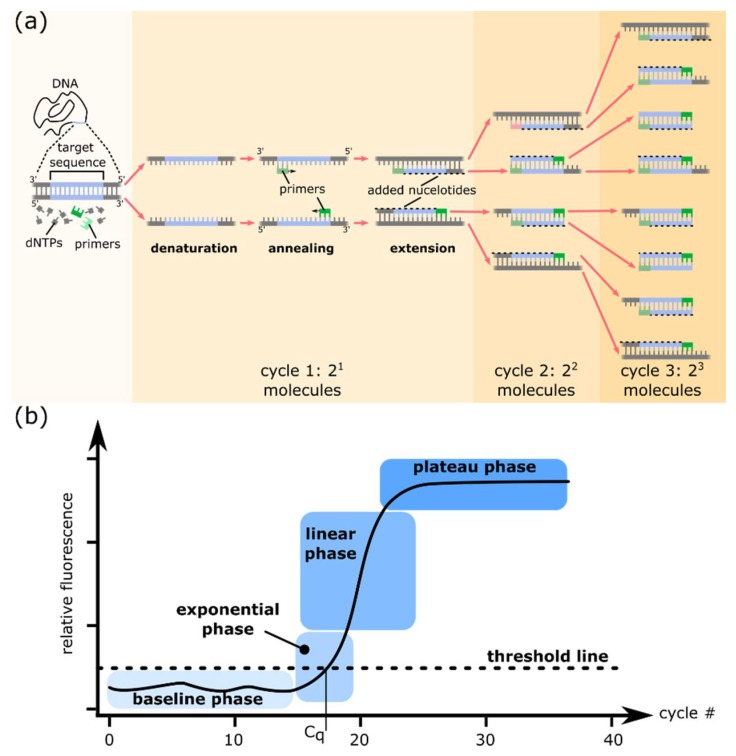
(**a**) Principles of the polymerase chain reaction (PCR). Each PCR cycle includes three steps: (1) Denaturation of double-stranded DNA by heat; (2) Annealing of primers to their complementary target DNA sequences; (3) Extension of primers by a thermostable DNA polymerase. A typical PCR reaction is cycled 20–40 times. Each cycle can theoretically result in a doubling of the number of molecules of the target sequence; (**b**) Different phases of a real-time PCR amplification plot on a linear scale. A typical PCR amplification plot displays a sigmoidal-shape curve with 4 distinct phases. (1) In early PCR cycles, the fluorescence signal due to amplification product remains at background level. The baseline is set to eliminate the background fluorescent signal. (2) During the exponential phase, the amount of PCR product doubles with each cycle in perfect reaction conditions, i.e., if amplification efficiency is 100%. The threshold (dotted lines) is set above the background within the exponential phase. The cycle of quantification (Cq) is the cycle number at which the amplification plot intersects the threshold line that is set significantly above the baseline. (3) The linear phase indicates that reagents are becoming limited, which results in a reduction of the amplification efficiency. The amplification signal is no longer exponential. (4) The plateau phase indicates a saturation of the signal. Reagents are depleted and no additional PCR product is generated or detected.

Polymerase Chain Reaction (PCR) is an in vitro technique that amplifies DNA, generating several millions of copies of a specific segment of DNA from a minute amount of starting material [1]. Its specificity relies on sequence hybridization and its sensitivity depends on enzyme-based amplification. PCR typically consists of a series of temperature cycles repeated 20 to 40 times. Each cycle includes the denaturation of DNA duplexes, the hybridization of two DNA oligonucleotides (primers) flanking the target sequence, and the elongation of those primers by a DNA polymerase (Figure 1a). Each cycle results in a doubling of the number of target DNA molecules (exponential amplification) and 2^n^ copies can, in theory, be produced after n cycles. In practice, the amplification process saturates and reaches a plateau as PCR reagents are depleted and accumulated PCR products self-anneal, preventing any further amplification. Conventional PCR analyzes amplified products at the end of the reaction using gel electrophoresis (end-point measurement).

**Figure 2:**
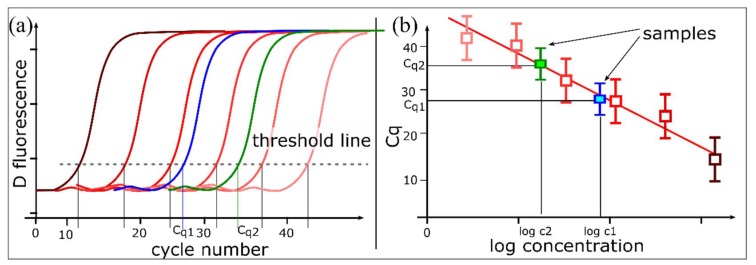
Real-time qPCR assay using a standard curve. (**a**) Amplification curves for a 6-point 10-fold dilution series of a template with known concentrations (standard) over five orders of magnitude (e.g., genomic DNA, PCR amplicon, linearized plasmid). The Cq value of each serially diluted standard is determined; (**b**) A standard curve is generated by plotting the Cq values derived from the amplification curves of the dilution series against the logarithm of the standard quantity. The standard curve is used to interpolate the quantity of the target. The slope of the standard curve measures the amplification efficiency of the qPCR assay. A slope of –3.32 (for a standard curve generated from a serial 10-fold dilution series) indicates 100% amplification efficiency, i.e., the amount of PCR product doubles during each cycle.

Real-time PCR is based on PCR and measures the amount of PCR product after each round of amplification using a fluorescent readout [2]. A typical real-time PCR amplification plot displays a sigmoidal-shaped curve (on a linear scale) and includes a baseline phase, followed by an exponential phase that reaches a plateau via a linear phase (Figure 1b). The exponential phase represents the most efficient phase of amplification and the amount of PCR products doubles with each cycle if the amplification efficiency is 100%. Real-time PCR enables the relative quantification of a target to a calibrator. The method is quantitative (qPCR) when calibrated with a standard curve using data from the exponential phase. The ‘absolute’ amount of target sequence in a qPCR reaction is measured relative to a standard curve generated from a sample of known quantity or copy number (Figure 2). This method implies that the amplification efficiencies of the sample and the standards are equivalent. Differences in PCR efficiencies can significantly affect the quantification accuracy [3].

While qPCR may be labor-intensive and suffers from limited reproducibility [4,5,6,7]; it is widely implemented in clinical settings and remains the gold standard for nucleic acid quantification.

### 1.2. Fundamentals of dPCR

**Figure 3:**
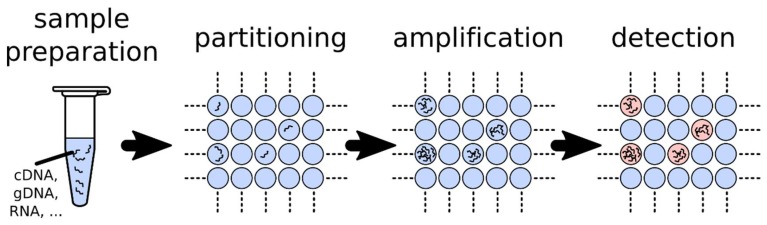
Principles of digital PCR. The sample is divided into many independent partitions such that each contains either a few or no target sequences. The distribution of target sequences in the partitions can be approximated with a Poisson’s distribution. Each partition acts as an individual PCR microreactor and partitions containing amplified target sequences are detected by fluorescence. The ratio of positive partitions (presence of fluorescence) over the total number allows to determine the concentration of the target in the sample.

Digital polymerase chain reaction (dPCR) enables the absolute quantification of target nucleic acids present in a sample and alleviates the shortcomings of qPCR [8,9,10]. In dPCR, the sample is first partitioned into many independent PCR sub-reactions such that each partition contains either a few or no target sequences (Figure 3). After PCR, the fraction of amplification-positive partitions is used to quantify the concentration of the target sequence with a statistically defined accuracy using Poisson’s statistics [11,12]. Interestingly, sample partitioning efficiently concentrates the target sequences within the isolated microreactors. This concentration effect reduces template competition and thus enables the detection of rare mutations in a background of wild-type sequences. Furthermore, it may also allow for a higher tolerance to inhibitors present in a sample.

### 1.3. Fundamental Differences between dPCR and qPCR

**Figure 4:**
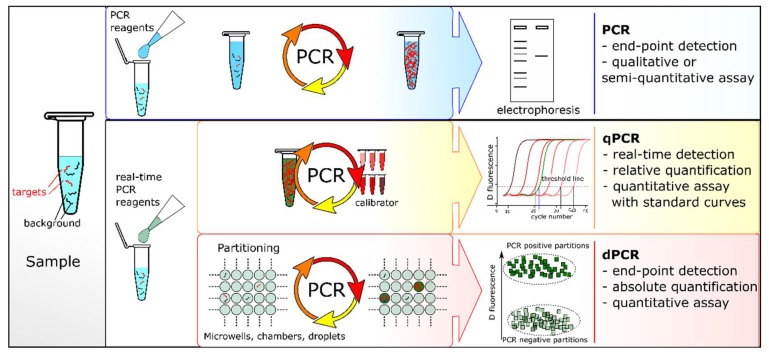
Comparison of PCR-based techniques. In conventional PCR, the amplification products are analyzed at the end of the reaction (end-point PCR) by gel electrophoresis and detected after fluorescent staining. qPCR and dPCR use the same amplification reagents and fluorescent labeling systems. In qPCR, the amount of amplified DNA is measured at each cycle during the PCR reaction, i.e., in real-time. The ‘absolute’ quantity of target sequence is interpolated using a standard curve generated with a calibrator. In dPCR, the sample is first partitioned into many sub-volumes (in microwells, chambers or droplets) such that each partition contains either a few or no target sequences. After PCR, the proportion of amplification-positive partitions serves to calculate the concentration of the target sequence using Poisson’s statistics.

The key difference between dPCR and qPCR lies in their strategy to measure the amount of target sequence. In qPCR, the reaction is monitored throughout the amplification process, and quantification is based on the analysis of the fluorescent signal at the exponential phase. In contrast, dPCR collects fluorescence signals via end-point measurement and uses the number of positive partitions over the total to back-calculate the target concentration (Figure 4). dPCR reduces quantification to the enumeration of a series of positive and negative outcomes thus converting a continuous or analog signal into a series of binary or digital signals. Unlike qPCR, dPCR does not rely on calibration curves for sample quantification. Hence, it avoids the pitfalls associated with variations in reaction efficiencies [3]. Quantification by dPCR is based on binomial statistics that mathematically define its inherent accuracy and performance metrics.

In brief, dPCR is a method of absolute nucleic acid quantification that hinges on the detection of end-point fluorescent signals and the enumeration of binomial events (absence (0) or presence (1) of fluorescence in a partition) (Section 2) [13]. This statistical foundation permits to identify the parameters that constraint the performance metrics of this analytical method (Section 3). dPCR is theoretically advantageous over qPCR given effective means to perform sample partitioning (Section 4) and target amplification of single molecules (Section 5 and Section 6). In practice, qPCR can still outcompete dPCR for specific applications thanks to higher sensitivity (Section 7).

## 2. Statistical Foundations of dPCR

dPCR benefits from statistical foundations that permit to infer both the target concentration and the accuracy of the quantification. This section reviews the statistical approaches underlying quantification by dPCR. Those approaches depend directly on the specific applications of the dPCR assay, *e.g.*, absolute quantification or copy number variant analysis.

### 2.1. Binomial Probability and Poisson Approximation

To estimate the probability *p* of a partition to contain at least one target sequence, we should consider the case of the random distribution of *m* molecules into *n* partitions. This situation corresponds to a binomial process where the outcome of each drawing is either present or absent and the drawing is repeated *m* times. The chance of a target sequence to be present in a partition is $\frac{1}{n}$ because it results from random or independent events. The probability *p* is the complementary chance of the partition to be empty after the *m* target sequences are distributed. A partition has *m* chances, or attempts, to receive one target sequence. The chance for a partition to be empty is then $1-\frac{1}{n}$ after one drawing, and ${\left(1-\frac{1}{n}\right)}^{m}$ after *m* attempts, finally $p=1-{\left(1-\frac{1}{n}\right)}^{m}$. In the situations where n is large ($\frac{1}{n}$ very small), one can consider the term $\left(1-\frac{1}{n}\right)$ as the first order approximation of $e^{-\frac{1}{n}}$, hence the probability *p* can be approximated to $p=1-\;e^{-}$ where $\lambda =\;\frac{m}{n}$. This formulation defines the probability function of a Poisson distribution of parameter lambda. Poisson distribution describes the probability distribution of independent events where the average number of events (λ) is known. The Poisson distribution predicts the proportion of partitions containing a given number of target sequences. Conversely, knowing the distribution permits to calculate the average number of a target sequence in the sample. Even though target partitioning follows a Poisson distribution, dPCR does not provide a detailed distribution and only indicates whether target sequences are present or not in a partition. Nonetheless, the ratio of positive partitions *k* (containing some target sequences) over the total number of partitions *n* is sufficient to predict the initial concentration of the target sequence in the sample with $\lambda =-\ln\left(1-\;\frac{k}{n}\right)$.

### 2.2. Quantification Accuracy

Intuitively, the confidence in the estimation of the target concentration depends on the number of empty partitions. In extreme cases, i.e., when most of the partitions are either empty or full, the confidence in the estimated concentration is very low because the pattern empty/full is not very informative.

The confidence interval is typically estimated using functions that can be directly calculated. Those estimations rely on assumptions that have direct consequences on the estimation. For instance, the Wald method approximates the binomial distribution (a discrete function with finite support) with a normal distribution (a continuous function with infinite support) [14,15]. As already noted [16], this approximation provides inaccurate results if most of the partitions are empty or if more than half of the partitions are filled. The Wilson method or interval [17] is thus preferred for direct calculation. In this case, the confidence interval is given by:

$$\left(p+\frac{\alpha^{2}}{2n}\pm \alpha \sqrt{\left(\frac{p\left(1-p\right)}{n}+\frac{\alpha^{2}}{4n^{2}}\right)}\right)/\left(1+\frac{\alpha^{2}}{n}\right)$$

where *p* is the probability that a partition is empty, *n* the total number of partitions and α is equal to 1.96 for a 95% confidence interval. Other methods, including the direct or Clopper-Pearson method, demonstrate better approximation but the equations must be numerically solved [14]. Furthermore, these numerical-based methods are rarely used for dPCR [16].

**Figure 5:**
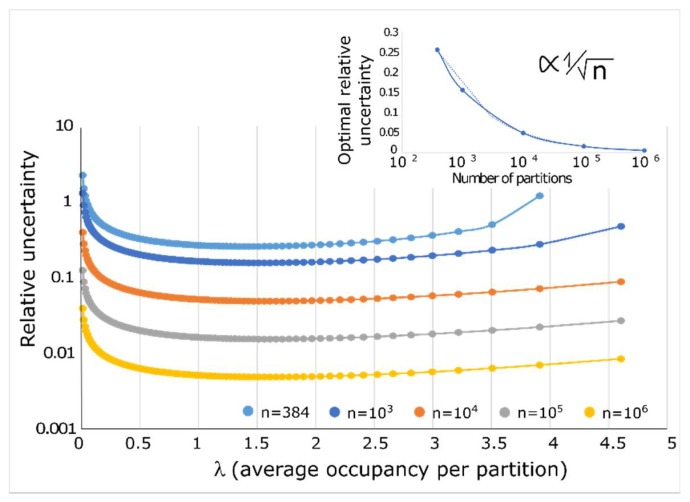
Quantification accuracy of dPCR. The precision of dPCR is non-uniform and depends on the average occupancy of target sequence per partition. The precision of dPCR also increases with an increasing number of partitions (distinct colors). The inset shows that the evolution of the relative uncertainty (taken at λ ≈ 1.6) decays as an invert square root of the number of partitions.

The previous considerations suggest that there exists a value of lambda for which the initial template concentration can be estimated with the highest confidence. In cases of 10,000 or more partitions, the maximal confidence is obtained for a λ value of about 1.6, which corresponds to a proportion of 20% of empty partitions (Figure 5). As noted previously, the precision is poor for low values of λ, reaches an optimal for a λ of 1.6 before slowly declining with increasing values of λ, which corresponds to a saturation of the partitions. The accuracy of the estimation of λ increases with the number of partitions and the optimal precision (at λ = 1.6) scales as the inverse square root of the number of partitions (Figure 5, insert).

### 2.3. Most Probable Number (MPN)

For over a century, digital assays were conducted to estimate the concentration of microorganisms of public health concern [18,19,20,21]. These estimations were based on repeatedly sampling a specimen at different dilutions to optimize the chances of estimating the concentration of microorganisms with the greatest confidence. However, such methods take into account the values from the entire dilution series and treat the concentration of the target as a parameter to optimize the probability of observing those experimental values (method of maximum likelihood) [22]. The probability function can be numerically optimized with various approaches, which gives rise to different MPN methods. The values are usually tabulated according to the dilution ratios, number of samples and estimation strategies [23]. Those MPN methods provide comparable results to the Poisson approximation while being more cumbersome to implement in digital PCR applications [16,24]. However, the MPN method is the appropriate approach when analyzing multi-volume dPCR [25,26].

### 2.4. Copy Number Variant (CNV) Applications

dPCR has been extensively used to measure genetic imbalances, or Copy Number Variant (CNV), that result from the deletion or amplification of genomic regions or locus. In CNV analysis, the copy number of a locus relative to another is the relevant information. The statistics to estimate the presence of a genetic imbalance using dPCR employs various methods. One such method relies on the Sequential Probability Ratio Test (SPRT), initially developed for quality control. It continuously tests two concurrent hypothesis while accumulating data until a hypothesis is considerably more probable than the other [27]. In dPCR, SPRT was used to distinguish between homozygosity and heterozygosity in specific cell types in the presence of a homozygous background [28,29,30,31,32]. Other studies directly considered the statistical analysis of the ratio of two λ estimated for two loci [33]. The ratio of λ was log transformed to normalize its distribution and to enable the derivation of its confidence. Alternatively, the confidence interval of the ratio was derived with an algorithm based on Fieller’s theorem [11].

### 2.5. Absolute Limit of Quantification Due to Specimen Sampling

The analysis is only performed on a sample, which is a small portion of a specimen. This imposes a fundamental limit on the quantification accuracy. The concentration of an analyte in the tested sub-volume may differ greatly from the concentration of the analyte in the entire specimen due to statistical sampling [34,35]. In other words, even a perfect quantification cannot properly determine the true concentration of the analyte in the specimen. The variability between samples can also be estimated using a Poisson distribution. For instance, if the average number of the target sequence in a sample is 1, the chance of quantifying the true concentration in any sample is only a third. This highlights two critical aspects: (1) low values of λ are not correctly quantified, which is a fundamental inaccuracy that exists for all sampling techniques; and (2) this sampling error or noise is not systematic but random and can be only reduced by analyzing multiple samples of a same specimen.

### 2.6. Hypothesis and Technological Implications

Poisson statistics relies on two assumptions: (1) target sequences are randomly distributed across partitions and (2) all partitions have the same volume. The random distribution of target sequences has been validated experimentally by deriving the Ripley’s K function that measures the randomness of the spatial distribution of partition occupancy [36,37], or by confirming that the estimated concentrations using sub-arrays are consistent [16]. However, precautions are necessary when quantifying target sequences localized in same genomic regions. For instance, this is the case when estimating the copy number of the HER-2 oncogene that amplifies within a short region of chromosome 17 [38]. If the target sequences are not physically separated, they end up in the same partition and lead to an underestimation of the gene copy number in the sample [33]. Conversely, the assumption of random distribution has also been used to measure the linkage of different genes by dPCR [39]. This approach relies on the multiplexed detection of different targets that produce specific fluorescent signals. The co-amplification of two target sequences in the same partition produces a dual-colored signal that indicates their presence on the same DNA template. Interestingly, linked targets (i.e., a single molecule with two different target sequences) have been used to assess the prevalence of molecular dropout i.e., the absence of amplification despite the presence of a target sequence in a partition [12]. The rate of molecular dropout was estimated from the number of single color partitions compared to the number of two-color partitions.

Quantification by dPCR assumes that partitions possess identical volumes; however, a large degree of variability in volume may be observed. The latter directly depends on the methods used to create the partitions. The effect of this variation in partition volume has been experimentally assessed and considered a potential source of dPCR imprecision [36]. For λ higher than 1, an increased variance in partition volume results in the over-estimation of empty partitions. In this situation, the proportion of empty partitions is lower than the proportion of partitions that contain a single molecule. As a result, the number of partitions that should be empty but capture a molecule due to a volume increase is lower than the number of partitions that should contain 1 molecule but end up empty due to a decrease in partition volume. In contrast to statistical uncertainty, the effect of partition volume variability on quantification accuracy does not decrease with the number of partitions. This inaccuracy will thus dominate the dPCR imprecision at high number of partitions [40]. A theoretical analysis concluded that the variability of the partition volume has a minimal impact when it is below 10% or when λ is lower than 1, but its effect should be considered otherwise [41]. Volume variation of commercial systems have been reported to be lower than 3% [42], but research prototypes can suffer from high volume variation depending on the type of fabrication used and on the physical principles underlying the partitioning step.

### 2.7. Conclusion of the Statistical Foundations of dPCR

dPCR is a statistical method that divides a sample into numerous partitions and enables the enumeration of empty and occupied partitions to determine the concentration of a target sequence present in a sample. dPCR is an absolute quantification method that does not rely on calibration curves and whose accuracy is more easily predictable [41]. The theoretical foundations of dPCR are well established but it is critical to appreciate the inherent statistical limitations of this method. The precision of dPCR is limited by the uncertainty of the measurement due to: (1) specimen sampling, whose effect is prevalent for low target concentrations and can only be minimized by using technical replicates; (2) its statistical nature whose effect on precision can be reduced by increasing the number of partitions [40]. dPCR’s intrinsic precision is not constant across its dynamic range and can be quite poor at the extremes. This is the case when most of the partitions are either positive or negative. Another technical limitation of dPCR stems from the variation in partition volume, which can have a detrimental effect at high average occupancy λ and can dominate quantification uncertainty at very high number of partitions. Those statistical considerations highlight the importance of the number of partitions, their volume and the standard deviation of their volume [41].

## 3. Performance Metrics

### 3.1. Sensitivity of Detection

The sensitivity or lower limit of detection corresponds to the detection of a single molecule in a single partition. Hence, the minimal concentration that can be detected depends on the total volume of the reaction or on the number of partitions and their volume. This simple reasoning underlines the limits of sensitivity of dPCR because dPCR techniques rely on partitions with volumes in the pL-nL range and their number is limited in practice. By contrast, the reaction volume of qPCR is typically much larger and can also be easily adjusted to reach higher sensitivity.

### 3.2. Dynamic Range of Detection

The dynamic range of detection is defined by the difference between the highest and the lowest detectable concentration of a molecule. The highest molecule concentration directly depends on the partition volume, i.e., partitions with smaller volumes correspond to higher molecule concentrations for a given λ. Interestingly, the highest number of target sequences detected can be far greater than the number of partitions. This value is estimated by solving λ for a given precision and number of partitions in the situation of high partition occupancy. For instance, given a precision of 12.6%, the highest number of target sequences detected can be 5-fold greater than 20,000 droplets generated [40], or 11-fold greater than 10^6^ partitions created [37].

From those considerations, a large dynamic range of detection creates opposing constraints on the volume of partitions; with larger partition volumes improving the lower detection limit and smaller partition volumes improving the higher detection limit (Table 1). This conundrum can be addressed by using dPCR designs with multi-volume partitions, where a series of large volume partitions assure high sensitivity while a series of small volume partitions allows high detection limit, and a few series of partitions with intermediate volume provide high precision [25,26]. Interestingly, this approach is equivalent to performing a series of different dilutions followed by a quantification using the MPN method. Furthermore, multi-volume dPCR allows to uncouple dynamic range and measurement precision [25]. On a practical aspect, this approach reduces the overall number of partitions required to reach a given dynamic range and hence the overall footprint of devices. 

### 3.3. Practical Considerations in the Reliability of dPCR Measurements-False-Negative/Positive Signals

Sensitivity is highly dependent on the rates of false positive and false negative events. Although dPCR is a digital assay, the signal detected is initially analog and a threshold needs to be applied to separate true signal from background signal (see [43] for a statistical thresholding method). False positives can arise from poor assay design or from the detection of spurious amplification at high number of PCR cycles. Additionally, they may also stem from cross-contamination during experimental set-up [40].

While false positives can be minimized by proper assay design and optimization [44], false negative or molecular dropout are less tractable. The intrinsic design of dPCR assays makes it prone to molecular dropout for various reasons: (1) the increased surface to volume ratio due to the small volume (pL-nL range) partitions increases the chance of PCR inhibition due to interactions of the reagents with surfaces or interfaces [45]; (2) it has been observed that single molecule amplification is often less efficient than amplification with higher number of molecules [36]; (3) the amplification efficiency is highly dependent on the source of DNA (i.e., genomic vs. plasmid, fragmented vs. long DNA molecules) [12,36,42], and can be impaired by exposure of DNA molecules to heating [46].

The mathematical framework introduced previously covers the statistical nature or intrinsic uncertainty of dPCR; however, the exact variance of dPCR assays should include the effect of upstream processes such as DNA extraction and pre-amplification [47]. For instance, it could be tempting to pre-amplify a sample with low target concentration to reach the optimal λ value of 1.6. However, the variance associated with the pre-amplification reaction is not systematic and cannot be corrected. As a result, direct quantification of low target concentration is still preferable [12,48].

The sensitivity of dPCR to molecular dropout or the variability of the sample preparation (extraction and/or pre-amplification) needs to be considered when assessing assay accuracy. Proper assay design and validation are critical to minimize typical issues arising from molecular dropout, false positives, or poor signal thresholding [13,44].

## 4. Miniaturization and Hyper-Compartmentalization

### 4.1. Introduction

Although the recent development of dPCR has been supported by advances in device miniaturization, the concept of dPCR has been developed [9,10] using microtubes [9] or 384-well microplates [10,28,29,30,31,49]. These formats suffer from limited number of partitions, limited automation and from the cost associated with the large amount of reagent needed. Microfluidics, i.e., miniaturization of fluid-handling [50], has enabled the massively-parallel sample partitioning and the advent of dPCR platforms. Microfluidics relies on microfabrication techniques adapted from microelectronics and its implementation relies either on fast prototyping by soft lithography in Polydimethylsiloxane (PDMS) [51], glass etching [52], or injection molding [53]. Numerous active and passive microfluidic methods have been used to compartmentalize samples, from physical partitions to liquid droplets. Most of those methods allow for simple automation and limited reagent use.

Before reviewing the different principles and methods employed to create partitions, it is worth mentioning some partition-free approaches. For instance, an early approach utilized a fused-silica capillary, typically used for capillary electrophoresis, as a reaction vessel to perform PCR on diluted DNA molecules. The number of amplified molecules was counted after electro-migration using an inline fluorescence detector [54]. This strategy relies on the limited diffusion of the amplicons generated, which migrate altogether as a plug during electrophoresis. The signal is a succession of peaks that corresponds to the number of target sequences in the sample. A more recent approach is based on the transformation of target sequences into 1 μm DNA nanoballs by Rolling Circle Amplification (RCA) [55]. The DNA nanoballs can then be enumerated under a microscope or a microfluidic cytometer. 

In the following section, we distinguish physical partitions where the reaction is partitioned into isolated chambers or microwells from droplet emulsions that can be collected outside the microfluidic devices. 

### 4.2. Chamber Formats

Performing dPCR with physical partitions or chambers involves device filling, sample partitioning, thermocycling and assay readout. We differentiate active partitioning methods that involve either device reconfiguration or mechanical actuation from passive partitioning methods that are driven by fluidic effects or properties. We further distinguish self-partitioning methods that include both passive filling and partitioning.

#### 4.2.1. Active Partitioning Platforms

**Figure 6:**
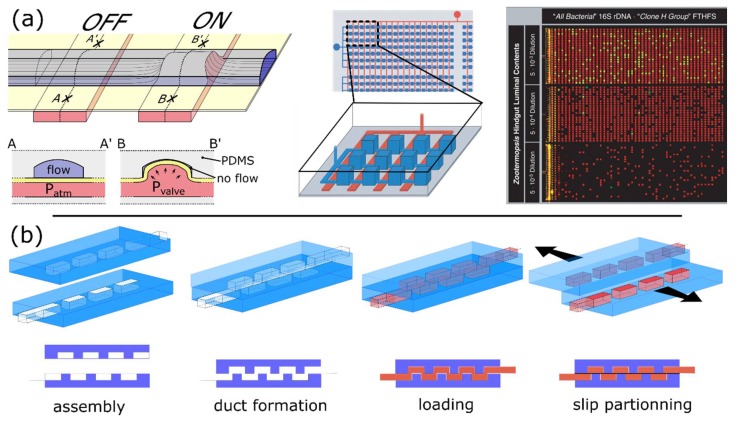
Active partitioning platforms. (**a**) Schematic of a push-up valve in a microfluidic chip made of PDMS. Left panel: The control channels (red) are separated from the fluidic channels (blue) by a thin flexible membrane (yellow). The flow through the fluidic channel is unobstructed if the control channel is not pressurized (OFF valve). When the control channel is pressurized, the thin PDMS membrane deforms and bulges into the fluidic channel, obstructing the flow (ON valve). As depicted in the bottom panel, a tight seal is obtained if the top of the fluidic channel is rounded. Central panel: Schematic diagram showing many parallel chambers (blue) connected through channels to a single input. The network of control channels (red) creates valves between each chamber allowing the partitioning of their content into independent PCR microreactors. Right panel: Three panels of 1176 chambers each, show the results of dPCR on samples harvested from a single termite (*Z. nevadensis*). (Figure adapted from [65] with permission.); (**b**) SlipChip device relies on two chip halves that contain arrays of open chambers. Temporary ducts or channels are formed when the two parts are aligned and put in contact while submerged in mineral oil. Sample and reagents are injected before the device is reconfigured by slipping. The slipping motion creates arrays of independent microreactors. The device can be further slipped to bring the two independent arrays (containing reagent and sample) in contact to trigger mixing (not shown). (Figure adapted from [66] with permission.)

One of the first microfluidic dPCR device relied on microfluidic valves that were created by superimposing a fluidic and a control networks of microfluidic channels made of the elastomeric material PDMS [56]. Those networks are separated by a thin membrane that can be deformed into a microfluidic channel by applying pressure into the opposing control channel to create an “on-off” valve (Figure 6a). The workflow includes: (1) filling all the chambers with the reaction; (2) pressuring the control layer, which closes the connection between the chambers, thus isolating the chambers from one another. Such a device enabled the creation of 14,112 × 6.25 nL partitions. The volume variation of the partitions depends on the precision of the soft-lithography process [51] used for the microfabrication.

The SlipChip platform [52] also uses an active partitioning approach (Figure 6b). The device is composed of 2 chip halves, each etched with two independent arrays of microwells [16]. The chip is assembled by putting into contact and aligning the two open-faced halves such that the chambers from the opposite halves form temporary continuous serpentine microfluidic channels. The sample and reaction mix are then flowed through independent microfluidic networks and are subsequently compartmentalized into arrays of independent chambers by slipping the chip halves. Further slipping assures superimposition of the sample and the PCR arrays creating a single array of independent microreactors. The chip is assembled in mineral oil, which lubricates the system during slipping and ensures the isolation of partitions. Partitioning is effectively achieved by mechanical shearing applied during the slipping motion. This strategy enabled the creation of 1280 partitions of 2.6 nL without the need for pumps and valves. Additionally, the authors mention that they could create up to 16,384 microwells of picoliter volume using the same footprint [16]. 

#### 4.2.2. Passive Partitioning Platforms

Passive partitioning uses fluidic effects to create sub-volumes and does not rely on mechanical methods. Arrays of microwells have been used to create partitions with either active or passive methods. This format can be considered as a direct miniaturization of a 384-well microplate, where the volume of individual microwells ranges from pL to nL [57,58]. The key difference from its macroscale counterpart is that microwells are usually loaded all at once to fully exploit the parallelization offered by the format. This in turn necessitates a method that isolates microwells from one another and avoids rapid evaporation of minuscule volumes. To support efficient microwell filling and partitioning, it is necessary to have differential surface properties between the interior of the microwell that needs to be hydrophilic and the top face of the array (in between the microwells) that needs to be hydrophobic [58,59,60].

The open version of the array of microwells has been the foundation of both active and passive partitioning platforms. Partitions were actively created by injecting the aqueous phase in the microwells, which were pre-layered with an immiscible oil, using a microdispenser [61]. Alternatively, the partitioning can be performed by the apposition of a glass slide [62], a deformable membrane [63], or pressure-sensitive tape [64] after assay loading.

In contrast to these active strategies, partitioning with this format can be performed passively by using an overlay of immiscible oil after loading of the aqueous phase into the microwells. The oil phase preferentially wets the top of the array and creates a meniscus that displaces the aqueous phase; however, the oil/aqueous phase/solid triple line gets pinned at transitions between hydrophobic and hydrophilic areas [58,67]. The liquid-liquid interface then extends from the pinned triple line until it reaches another hydrophobic patch where another propagating triple line will be created [68]. The oil progresses on the hydrophobic surface of the array and around the well orifices to generate a sweeping motion that displaces the excess of aqueous phase.

**Figure 7:**
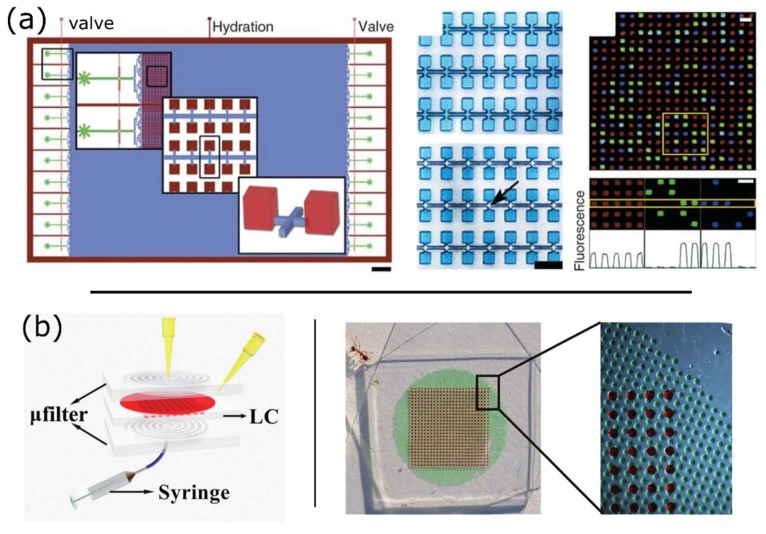
Passive partitioning platforms. (**a**) Megapixel digital PCR using planar emulsion arrays. **Left panel**: Schematic of the megapixel digital PCR device, with insets showing the array and chambers at increasing magnification. Scale bar: 3 mm. Central panel: The chambers are loaded with a blue dye. The arrow indicates chambers isolated by immiscible oil. Scale bar: 50 μm. Right panel: Multiplexed detection of *HLCS* (green) and *RPPH1* (blue) over 342 chambers. (top). A close-up view shows the signals of the boxed chambers in the different fluorescence channels (middle), with the corresponding intensity profile of the middle row (bottom). Scale bars: 50 μm. (Figure adapted from [37] with permission.); (**b**) Vacuum-assisted reagent loading in a PDMS-based array of microwells. Left panel: The schematic of the microfluidic chip depicts the process of reagent loading into the lamina chip layer *via* a μfilter layer. The syringe is used to create a temporary vacuum through the PDMS layer and drive liquid into the microwells. Right panel: Device loaded with the reagent (red) and water (blue). (Figure adapted from [72] with permission.)

Pinning also exists when a triple line encounters an abrupt change of topology or channel direction [69,70]. Pinning can thus be used to isolate dead-end chambers within a microfluidic network thanks to the topology of the main channel and the chambers (Figure 7a) [37]. In this configuration, the oil film gets pinned at the chamber orifices. This strategy greatly increases the chamber density by reducing the size of the main channel compared to microfluidic valves that require a minimum span or width to be efficiently deformed. This method resulted in the generation of up to 1 million partitions in the pL range with a standard deviation of the volume equal to a few percent [37]. The very high number of partitions allows unparalleled precision and a theoretical dynamic range of up to 7 logs. In addition to a much higher density of chambers, the strategy requires a simpler fabrication process than pneumatic valves.

Critically, the filling of chambers at small scale is not trivial and is constrained by capillary effects [68]. In practical terms, the injected liquid needs to let the air exit the volume in a coordinated fashion. This requirement is alleviated using PDMS, an elastomer permeable to gases. Chambers are filled by pushing the air out through the material, by pressuring the incoming liquid. Alternatively, the device can be packaged under vacuum [71] or vacuum can be applied to a chamber located underneath an array of microwells to drive filling (Figure 7b) [72]. This approach avoids the risk of losing the sample through a small leak. PDMS presents however several drawbacks: (1) DNA and protein tends to absorb onto its hydrophobic surface if not properly pre-incubated with a solution of BSA [67,73], which in turn may affect its surface properties; (2) it is permeable to water and evaporation must be mitigated by incorporating water reservoirs [74] and vapor barriers made of parylene C [37] or glass [43], which complicates device fabrication; (3) it suffers from a high cost of production, which impedes its use in large-scale manufacturing. 

#### 4.2.3. Self-Digitization Platforms

**Figure 8:**
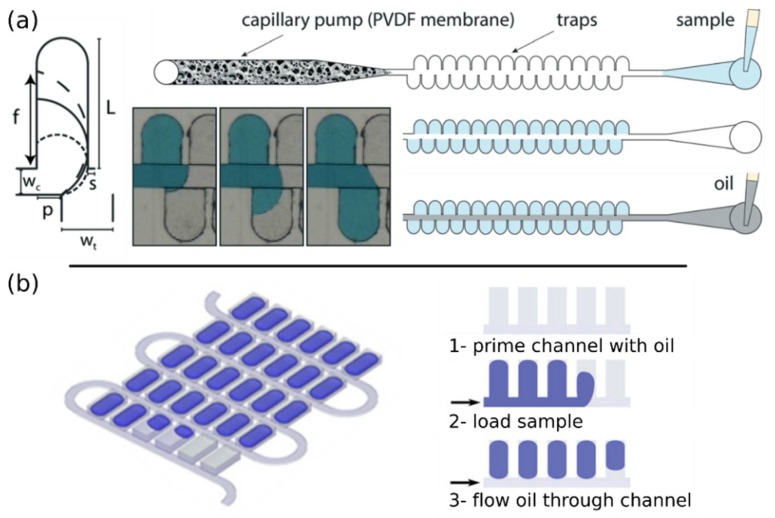
Self-filling and partitioning platforms. (**a**) Geometry for a staggered trap configuration where filling is controlled by pinning. The design enables an efficient filling by the sweeping motion of the solution through the traps and thus avoids trapping air within the chambers. The pinning offset is different on each side of the main channel thanks to a barrier wall. The loading process of the staggered device is shown in the pictures. The device incorporates a capillary pump that passively aspirates the excess of liquid. (Figure adapted from [69] with permission.); (**b**) Schematic of the self-digitization device that contains 1020 wells. The device is first primed with oil, then filled with the reaction mix and injected with another stream of oil to create an array of droplets trapped in the structures. (Figure adapted from [76] with permission.)

Self-digitization platforms combine both passive filling and partitioning. Passive filling can be enabled by harnessing the pinning effect to efficiently displace the air with a liquid during filling. This has been achieved by staggering two series of chambers across a main channel (Figure 8a) [69]. In this contraption, the liquid alternately sweeps through the chambers without trapping air because one extremity of the interface is pinned thanks to a barrier wall. The staggered configuration is critical to allow alternate pinning between the two sides of the main channel. The wetting of the aqueous phase on the plastic surface is increased by the addition of a surfactant and glycerol, which facilitates the filling phase. This platform also includes a capillary pump that pulls the excess of liquid from the device and simplifies the actuation of the system. The partitioning is completed by injection of an immiscible oil phase. This proof of principle generated an array of 768 × 11 nL partitions with a volume variation of 12%.

The actuation can also play a key role in simplifying an experimental set-up. For instance, spinning can distribute fluid into chambers located along a spiraling channel [75]. Unfortunately, this format does not permit a direct observation of the filling and partitioning steps, which would be useful to improve the channel design. Overall, this platform generated a series of 1000 × 33 nL partitions but with a volume variation of up to 16%.

In the self-digitization approach [60,74], the device consists of a main channel with side chambers (Figure 8b) [76]. The device is first primed with immiscible oil that wets the channel and chamber walls. The aqueous sample is then injected followed by another plug of oil to create partitions. The filling involves the displacement of the immiscible oil by the aqueous solution and requires the walls to be hydrophobic, which may appear counterintuitive [60]. The hydrophobicity of the wall assures the presence of a thin film of oil at its surface [77], which acts as the draining conduit during the phase displacement. In the case of hydrophilic walls, the aqueous phase interacts strongly with the walls and creates a plug that prevents the oil from leaking out of the chambers.

The process contrasts with passive partitioning because it involves the formation of droplets that are generated through the splitting of a plug through a network of chambers [78], and it does not rely on pinning and differential surface properties between the main channel and the chambers. Droplet splitting is indeed governed by the capillary number that characterizes the relative effect of viscous and capillary forces [79,80]. The partition volume is mostly set by the chamber volume, but it also depends on the geometry of the chamber, flow rate, capillary number, contact angle, and oil viscosity. A refined version of the self-digitization platform yielded arrays of 535 × 6 nL partitions with a partition volume variation of 10–15% [74]. The same group further applied the same principles to a network of microwells located at the bottom of a main channel [81]. This strategy yielded a higher density of partitions (38,400 partitions of 2 nL). It also enabled optimization of the droplet formation by adjusting the design of the main channel.

### 4.3. Droplet-Based Platforms

The first goal of emulsification is to create isolated microreactors of aqueous droplets within immiscible oil. A critical component of this technology is the surfactant and oil formulation that assures both the stability of those microreactors and their compatibility with molecular reactions such as PCR or isothermal amplification [82].

Encapsulation does not always perform sample partitioning and it can be used just to create independent microreactors such as in BEAMing (Beads, Emulsion, Amplification, Magnetics) [83,84,85]. In this method, partitioning is achieved with magnetic beads that capture the target sequences by limiting dilution such that a single molecule is captured per bead. Encapsulation is performed to generate single-bead droplets used as independent microreactors to amplify the target sequence and saturate the bead surface. Emulsions can be easily and quickly obtained by mechanical shearing, which generates polydisperse droplets. After bead recovery, bead-bound sequences tagged with a fluorescent label are identified by flow cytometry at very high throughput. The magnetic beads allow for a simple and efficient sample purification and manipulation. This approach is applied to quantify genetic imbalance of specific genetic loci [84,85]. The strength of this method resides in the transformation of a molecular signal into a cytometric readout with minimal constraints on the emulsification process.

**Figure 9:**
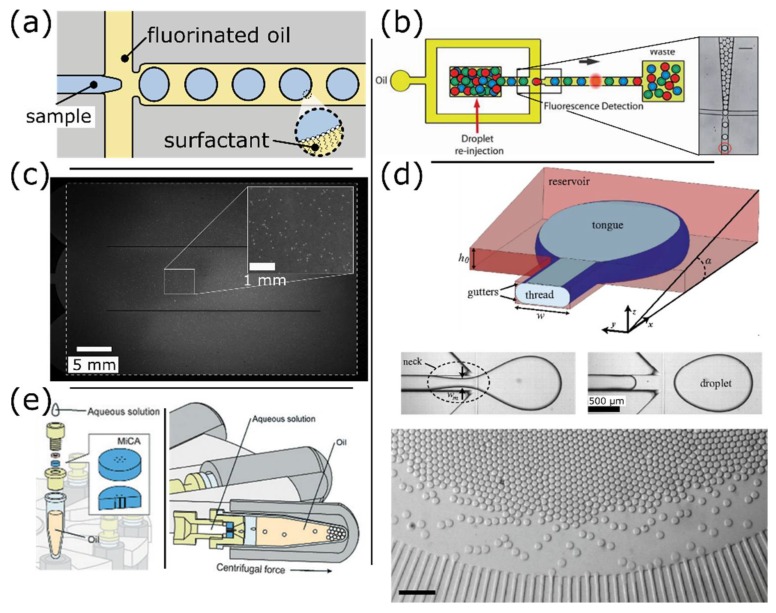
Microfluidic droplet-based platforms. (**a**) Schematic illustrating the generation of droplets with a nozzle or droplet generator. The aqueous phase is pinched by two streams of immiscible oil that stretch the interface via viscous forces until a capillary instability develops and the droplet detaches. Droplets are very stable thanks to surfactants that stabilize their interface; (**b**) The fluorescence signal from droplets can be sequentially detected in a single-file configuration. This arrangement is reminiscent of the optical and fluidic configurations used in flow cytometry. (Figure adapted from [86] with permission.); (**c**) Droplet signal can also be interrogated using a wide field detection that allows up to 1 million droplets to be analyzed simultaneously (Figure adapted from [43] with permission.); (**d**) Microfluidic droplets can be generated with a simplified experimental setup that relies on a gradient of confinement (a type of step emulsification). (Figure adapted from [87] with permission.); (**e**) Alternatively, highly parallel droplet generators have been adapted to actuation by centrifuge, which allows concurrent encapsulation of several samples. Scale bar: 500 μm. (Figure adapted from [88] with permission.)

Microfluidic droplet methods differ from BEAMing by using droplets as true partitions. They are enabled by microfluidic emulsification techniques that generate monodisperse droplets with very limited volume variation. Microfluidic droplets can be created with different techniques such as T-junction [89], nozzle (Figure 9a) [90], or step emulsification [91]. Droplet formation with T-junctions and microfluidic nozzles relies on the viscous shearing that overcomes capillary effects at the interface. Droplet generation via flow-focusing thus depends on the capillary number of the system. On the contrary, step emulsification is driven by an imbalance of Laplace pressure controlled by the geometry of the channel. Those droplet generation techniques result in the generation of streams of droplets with volumes ranging from pL to nL and a throughput of up to tens of thousands of droplets per second. In contrast to droplets generated in solid chambers, microfluidic droplets are not static but manipulated within networks of channels. Droplets can be collected off-chip for thermocycling and re-injected into a microfluidic device for readout. In droplet microfluidics, the sample does not interact with the channel walls once encapsulated, even though this may not preclude cross-contamination [40] or interfacial inhibition [92,93]. 

dPCR applications based on microfluidic droplets have been enabled by single molecule amplification [24,94,95,96,97]. Using droplet-based microfluidics, the number of partitions can be adjusted to meet the requirement of an application, with for example devices capable of generating over 1 million droplets [43]. Furthermore, the volume variation of microfluidic droplets resides within a few percent [42,89,90,91]. It also does not depend on the homogeneity of the microfabrication over a large array of features because all the droplets are usually generated using a single generator. This tight volume distribution remained lower than 3% when measured from droplets generated with 16 independent generators from five different eight-channel commercial cartridges [40]. The throughput of droplet generation can be increased with multi-nozzle systems [96] or through droplet splitting; [43] however, the effects of those techniques on the variation of droplet volume are unknown. Finally, multi-volume assays cannot be easily implemented in a single run using droplet microfluidics because the droplet size depends mostly on the nozzle dimensions, and manipulation of droplets in channels is complicated if droplets are polydisperse. 

The throughput of droplet digital PCR (ddPCR) is often limited by the readout that is typically performed by interrogating droplets sequentially in a configuration inherited from flow cytometry (Figure 9b). The readout throughput is lower than in cytometry because droplets cannot withstand high shear rates. This limitation can be overcome by converting droplets into cytometry-compatible particles such as magnetic [83,84,85,95,98] or agarose beads [98], or by using a double emulsion format [99]. Alternatively, a 3D particle counter (IC 3D) has been developed for rapid enumeration of positive droplets directly in the collection vial, which alleviates the need for further manipulation of the emulsion [100]. IC 3D is based on a horizontal microscope whose confocal volume scans the whole emulsion by rotating and moving the collection vial. More classic approaches include wide field detection strategies that have been implemented to image droplets arranged in 2D arrays or crystals (Figure 9c) [43,87]. This approach is cheaper and easier to implement, as it does not require any optical alignment. This format also permits real-time detection and melting-curve analysis, which provide efficient strategies to reject any spurious amplification that may be present at high number of thermal cycles. 

Minimizing the need for specialized equipment to perform partitioning represents an important technological trend. Microfluidic droplets have been generated using gradient of confinement, a method similar to step emulsification that permits to simplify actuation of the oil phase (Figure 9d) [87]. Interestingly, both step emulsificators [101] and droplet generators [88] have been adapted to actuation by centrifuges typically found in laboratories (Figure 9e). In addition to simplifying the set-up and streamlining the workflow, those approaches can increase sample throughput by enabling simultaneous encapsulation of multiple samples.

### 4.4. Conclusion of Hypercompartmentalization

A wide range of microfluidic approaches has been used to implement dPCR (Table 2). dPCR platforms aim at providing the optimal performance for dPCR by delivering a high number of partitions with limited volume variations and a large total reaction volume. dPCR technologies can be classified according to the format of the partitions and the methods used to create them. The partition formats include physical partitions and droplets. The principles underlying partitioning inform on the source of volume variation, the density of partitions that can be achieved and the simplicity of the set-up to perform partitioning. The basic principles involved in partitioning include: (1) direct mechanical shearing in microvalve-based arrays or in some cases of open arrays of microwells; (2) viscous shearing in the case of the SlipChip format and droplet generators; (3) pinning to control partitioning by immiscible oil in arrayed chambers or arrays of microwells, as well as to control passive filling of arrays of staggered traps; and (4) gradient of Laplace pressure to generate droplets.

Cost-effective manufacturing is also a critical factor for high volume or commercial applications, and plastic injection molding is preferred over PDMS. There is a trend towards simplifying the actuation, which will lower the cost of those platforms, reduce the hands-on time and support the widespread adoption of dPCR. dPCR platforms currently lack the sample multiplexing capability of qPCR.

## 5. Detection Methods and Multiplexing Approaches in dPCR

Similarly to qPCR, dPCR uses two main types of chemistries for the detection of nucleic acid: DNA intercalating dyes and hydrolysis-based probes [106]. Both detection methods generate a fluorescent signal that is proportional to the amount of DNA. DNA-binding dyes intercalate into double-stranded DNA (dsDNA). Upon interaction with dsDNA, DNA-binding dyes are stabilized into an excited state that results into a strong fluorescence. DNA-binding dyes are non-specific and interact with double-stranded DNA molecules irrespective of their sequence. By contrast, hydrolysis-based probes are sequence specific and different chemistries are used. In the 5′-nuclease methods, the fluorescent-labeled oligonucleotide probe is cleaved by the 5′ to 3′ exonuclease activity of the DNA polymerase after it hybridizes to the target sequence. The fluorescent reporter dye located at the 5’ end of the oligonucleotide probe is released and generates a fluorescent signal.

Multiplexed qPCR assays permit to simultaneously detect multiple targets in a single reaction using probes labeled with different fluorescent dyes. The number of target sequences that can be quantified concurrently by qPCR is thus spectrally limited by the spectral bandwidth of detection and the fluorescent characteristics of the dyes.

**Figure 10:**
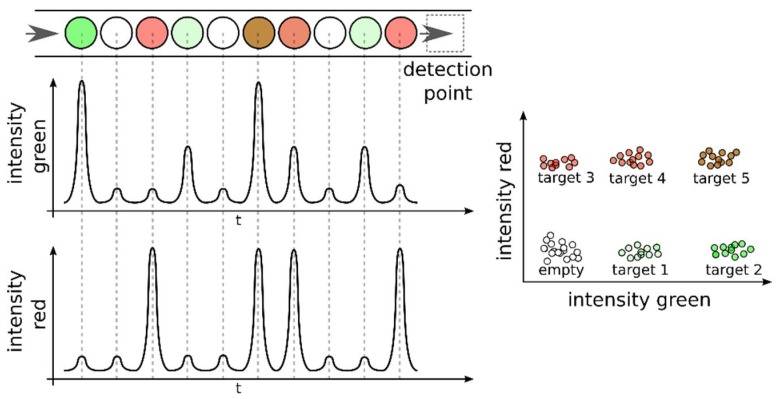
Multiplex droplet dPCR assays. dPCR assays can be multiplexed by coding the level of fluorescence of the plateau phase because dPCR can be implemented as a non-competing template assay, where each target is isolated in different partitions.

In multiplexed dPCR assays, the different target sequences can be coded not only with different fluorescent colors but also with different fluorescence intensities [104,105]. This strategy is enabled by the isolation of target sequences into independent microreactors combined with end-point detection (plateau phase of PCR). The total number of targets in a single reaction is a combination of the number of colors and the number of intensities that can be distinguished [102] (Figure 10). The fluorescent intensity of the signal is controlled via the concentration of the fluorescent-labeled probes [105,107]. Alternatively, DNA intercalating dyes can be used to differentiate amplicons of different sizes because the fluorescence generated is proportional to the number of molecules intercalated [103]. The multiplexing strategy in dPCR can be implemented with various schemes to address specific applications [108]. 

The sample can be encapsulated directly with the different assays in a one-step manipulation because dPCR avoids template competition. Multiplexed dPCR assays highlight the importance of robust algorithms for data thresholding and clustering [109]. Multiplexing using fluorescence levels assumes that different targets are isolated in different partitions. This ensures non-competing conditions and a simple correspondence between signal intensity and target identity.

## 6. Beyond PCR

### 6.1. Isothermal Amplification

Although PCR is established as the method of choice for molecular diagnostics, it presents several fundamental limitations such as high cost of equipment and sensitivity to inhibitors [110]. These limitations have led to the emergence of alternative nucleic acid amplification technologies (NAATS). Among them are methods that achieve nucleic acid amplification at a single reaction temperature obviating the need for thermal cycling. These isothermal amplification methods offer potential advantages over PCR-based approaches including speed, low cost, and simplicity of operation. Importantly, they exhibit an efficiency of amplification like that of PCR, and they promise a more manageable implementation in resource-limited settings.

A wide variety of isothermal nucleic acid amplification technologies have been developed such as rolling circle amplification (RCA) [111], nucleic-acid sequence-based amplification (NASBA) [112], loop-mediated amplification (LAMP) [113], multiple displacement amplification (MDA) [114], helicase-dependent amplification (HDA) [115], recombinase polymerase amplification (RPA) [116], strand-displacement amplification (SDA) [117], and exponential amplification reaction (EXPAR) [118,119]. The underlying chemical principles of the isothermal amplification reactions are specific to each method and may rely on the use of multiple primers and several enzymes. Furthermore, the use of a specific technology is determined by the user’s application. Recently, isothermal amplification in a digital format has been implemented using LAMP [101,120], RPA [66,74], RCA [55], MDA [121] and EXPAR [122].

### 6.2. Digital Isothermal Amplification Systems

A digital quantification system using RCA enables the detection of nucleic acids or proteins at the single-molecule level [55]. After circularization of target templates *via* ligation of padlock probes, the target templates are amplified using RCA and labeled with fluorophore molecule-tagged probes. The system was successfully applied for the quantification of *Vibrio cholera*, the causative agent of cholera. A digital RPA has been implemented on a SlipChip device allowing more than 1000 nanoliter-scale RPA reactions to occur simultaneously. The performance of the system was validated by detecting a single molecule of the methicillin-resistant *Staphylococcus aureus* (MRSA) genomic DNA [66]. SlipChip devices were also used for the quantification of viral RNA using digital reverse transcription-loop-mediated isothermal amplification (dRT-LAMP) [26,123]. Recently, a digital LAMP (dLAMP) SlipChip assay was developed to determine within less than 30 min the phenotypic antibiotic susceptibility of *E. coli* from urine specimens [120]. dLAMP was also implemented using the droplet format [124]. A digital MDA assay was reported to successfully investigate levels of DNA contaminant in sample preparations and commercial reagents [121]. EXPAR, which relies on the cooperative action of a DNA polymerase and a nicking enzyme, has been combined with the IC 3D detection system to quantify the amount of the Let-7a miRNA directly from plasma [122].

Some of these studies have compared the performance of digital isothermal amplification with dPCR. Digital MDA was found to be several orders of magnitude more sensitive than dPCR to quantify contaminant DNA. This result emphasizes the fact that MDA is not sequence-specific and unlike PCR, it does not require intact genomic DNA to generate a positive signal [121]. dLAMP was found to be less sensitive than dPCR for the detection and quantification of human cytomegalovirus (hCMV) [125]. The same observations were observed using droplet digital PCR [126,127].

In other instances, dLAMP and dPCR provided very similar results for the detection of *E. coli* DNA using a centrifuge-driven emulsification approach [124]. A dLAMP assay detected the presence of *E. coli* in urine samples within 7 min, while it required 2 h with droplet dPCR [120]. Despite its advantages, dLAMP relies on multi-step protocols that increase inter-assays variability. dRPA and dPCR performed on a SlipChip device exhibited comparable performance when quantifying MRSA genomic DNA [66]. However, RPA is sensitive to the presence of secondary structures and may require the use of chemical enhancers to disrupt those structures [66]. dPCR may also be preferred because reagents and template can be loaded onto devices as one mixed solution.

Overall, few studies have thoroughly compared digital assays based on isothermal amplification and PCR. This stems from the fact that isothermal NAATs and digital assays are both very recent technologies. There is a need for detailed evaluation and comparison of isothermal amplification with PCR in digital assays. Thus far, digital isothermal quantification does not necessarily demonstrate superior performance when compared to dPCR and have not fully delivered on their promise of simplified assays. Digital isothermal quantification relies on multi-step workflows, may require assays to be prepared at 4 °C to prevent spurious amplification or necessitate tight temperature control for optimal amplification [71]. Digital quantification is a relatively immature technology compared to that of qPCR. The field of isothermal amplification is rapidly evolving, and novel methods that can be transposed to a digital format will undoubtedly emerge.

## 7. Experimental Comparison of dPCR and qPCR

dPCR is expected to show higher resilience to inhibitors because target sequences are efficiently concentrated in smaller volumes. Several studies have reported the higher tolerance of dPCR to diverse types of inhibitors as compared to qPCR [44,125,126,128,129,130,131]. However, resilience to inhibitors depends specifically on the inhibitory agent [125]. It is thus unsafe to generalize, and each reagent and known inhibitors should be assessed thoroughly for its potential inhibitory effect [125]. dPCR may be especially useful for clinical specimens such as stool, sputum, and tissues known to contain many inhibitors [125,126]. Unexpectedly, a study reported that dPCR underperformed with clinical samples but not when using DNA standards [127]. However, the optimization of the assay was questioned [132].

dPCR outperforms qPCR in the analysis of copy number variation [13,33,105], and in the analysis of mutant abundance in viral [132] and cancer [133,134] studies. dPCR exhibited higher precision with a decreased coefficient of variation for the quantification of HIV DNA [135] or serum miRNAs [136] using droplet dPCR. In agreement with statistical considerations, dPCR precision depends on both the number of replicates and the template concentration [33]. Interestingly, RT-dPCR can reveal the unexpected variability in transcript levels of genes commonly used as references in RT-qPCR [137]. Concerns were also raised that the precision of dPCR could suffer from false positives in the quantification of HIV RNA [138].

A comparative evaluation of the performance between an established real-time qPCR assay and a droplet dPCR assay showed comparable detection sensitivity for the quantification of HIV-1 DNA with either droplet dPCR [139] or the SlipChip platform [66]. In other studies, RT-ddPCR and RT-dPCR showed less sensitivity than RT-qPCR at low viral load for the quantification of CMV RNA [125,127] or HIV RNA [138]. In contrast, RT-dPCR showed higher sensitivity than RT-qPCR for the detection of the biomarker *BCR-ABL* when combined with a pre-amplification step [140], but did not perform as well without pre-amplification [137]. The lower sensitivity of dPCR compared to qPCR is mostly attributed to the difference in the total reaction volume [13,125,127]. Interestingly, a side by side comparison between a dPCR assay performed on a Megapixel device and a qPCR assay revealed similar dynamic ranges [37]. These results are consistent with the fact that qPCR can be limited by non-template amplification in the lower range of concentration, and by the DNA-dependent inhibition of PCR in the upper range of target concentration.

The calibration-free nature of dPCR should confer an advantage on assay reproducibility [13]. In comparison, it is well documented that qPCR suffers from poor reproducibility [4,5,6,7]. Coefficient of variation are generally lower when using dPCR compared to qPCR; however, data comparing the day-to-day or inter-laboratory reproducibility of dPCR and qPCR are sparse. In one such study, ddPCR showcased much higher day-to-day reproducibility in the quantification of miRNAs in serum compared to qPCR [136].

Despite the relatively limited number of thorough studies that compare the performance of dPCR and qPCR side-by-side, a few key points emerge: (1) small partition volume contributes to dPCR resilience to a large variety of inhibitors; (2) dPCR is more precise for quantifying relative abundance (e.g., CNV, mutant allele burden); (3) dPCR suffers from lower sensitivity for absolute quantification, which is attributed to its smaller reaction volume. A key argument that could support the widespread use of dPCR in clinical settings would be its expected high technical reproducibility; however, day-to-day and inter-laboratory reproducibility of dPCR has yet to be rigorously assessed.

## 8. Conclusions

dPCR reduces the quantification of a target sequence to the enumeration of a series of positive and negative amplification reactions, thus converting a continuous or analog signal into a series of binary or digital signals. dPCR has been enabled by advances in microfluidics that provide efficient methods to create many independent reactors.

It is critical to understand the statistical foundations of dPCR to interpret data and appreciate the design parameters that define its performance. The key design parameters of dPCR platforms include the number of partitions, the volume of individual partitions, the total reaction volume and the variation of partition volume. The statistical precision of dPCR is further compounded by the variability of sample preparation and the rate of molecular dropouts and false positives. 

A wide range of microfluidic approaches has been used to develop dPCR platforms. The partitioning methods inform on the source of volume variation, the density of partitions that can be created and the simplicity of the experimental set-up. Through the rapid evolution of the platform technologies there is a clear trend towards simpler actuation, higher density of partitions, larger reaction volume, and the use of microfabrication methods that support high volume manufacturing.

dPCR platforms currently lack the sample multiplexing of qPCR while providing unique assay multiplexing capabilities. Isothermal amplification is an attractive alternative to PCR to reduce the complexity of the instrument and to further improve tolerance to inhibitors. Thus far, none of the emerging isothermal amplification techniques has exceeded PCR in terms of performance and assay workflow. Finally, even though only a limited number of studies directly compare the performance of dPCR and qPCR, it appears that dPCR is more resilient to inhibitors and provides higher precision for quantifying relative abundance (e.g., CNV) of target sequences. However, dPCR currently exhibits lower sensitivity compared to qPCR. 

dPCR is an emerging technology that may outperform qPCR in clinical applications thanks to its robustness and promised technical reproducibility. Microfluidic technologies have played a central role in enabling the revolution of digital quantification. They provided efficient methods to perform sample partitioning, which is at the core of the dPCR concept. Importantly, microfluidics is a very active field that keeps providing new and creative solutions to improve current platform performance.

## Figures and Tables

**Table 1:** Design parameters and performance metrics of dPCR.

Metrics	Accuracy	Sensitivity	Upper Limit
**Design parameter**	Number of partitions Variation in partition volume	Total assay volume or -Number of partitions × volume of partition	Volume of partition
**Comments**	Relative accuracy scales like $1/\sqrt{number\;of\;partitions}$ Variation in partition volume dominates inaccuracy at very high number of partitions	1/(Total assay volume)	Benefits from small partition volume

**Table 2:** Summary of partitioning platforms for dPCR.

Partitioning Method	Number of Partitions	Volume of Partitions	Principles	Reference
microfluidic valving	10^4^	10 nL	relies on the elasticity of the material	[56]
SlipChip	10^4^	10 nL	slipping for partitioning	[16,52,66]
open arrays of microwells	10^5^	10 pL	both active and passive strategies demonstrated	[61,62,63,64,67]
microfluidic chambers	10^6^	10 pL	pinning of the oil interface to isolate chambers	[37]
self-digitization	10^4^	10 nL	plug splitting within a network of chambers pre-wet with immiscible oil	[76,81]
self-filling	10^3^	10 nL	control of the pinned interfaces for controlled filling	[69]
spinning disk	10^3^	10 nL		[75]
droplet generator	10^4^–10^6^	10^1^–10^2^ pL	droplets used as partitions	[40,43,87,88,101,102,103,104,105]
